# A monoclinic polymorph of *N*,*N*′-bis­(2,6-diisopropyl­phen­yl)formamidine

**DOI:** 10.1107/S160053680802076X

**Published:** 2008-07-09

**Authors:** Jason D. Masuda

**Affiliations:** a Department of Chemistry, Saint Mary’s University, Halifax, Nova Scotia, Canada B3H 3C3

## Abstract

A new polymorph of *N*,*N*′-bis­(2,6-diisopropyl­phen­yl)formamidine, C_25_H_36_N_2_, is reported, which is different from the previously reported ortho­rhom­bic structure. The mol­ecule crystallizes in the *E*–*anti* configuration, with tautomeric disorder of the N-bonded H atoms and no clear distinction between imine and amine functionalities. The mol­ecules form hydrogen-bonded dimers with inter­molecular N⋯N distances shorter than those in the ortho­rhom­bic polymorph.

## Related literature

For the ortho­rhom­bic polymorph, see: Stibrany & Potenza (2006 ▶). For synthetic details and related literature, see: Krahulic *et al.* (2005 ▶); Perrin (1991 ▶).
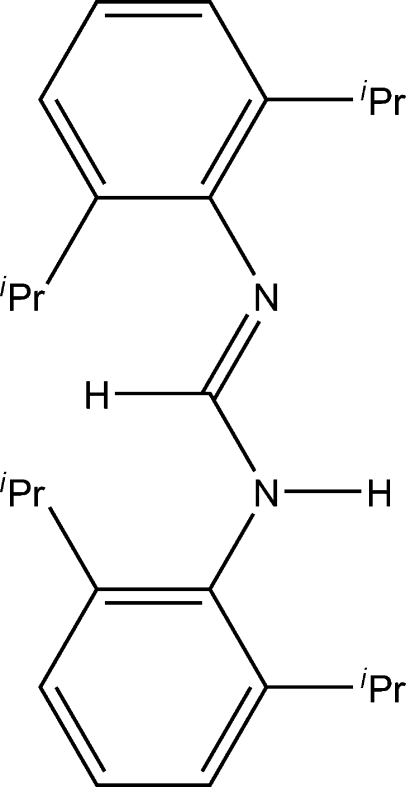


## Experimental

### 

#### Crystal data

C_25_H_36_N_2_
*M* *_r_* = 364.56
Monoclinic, 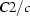

*a* = 24.169 (4) Å
*b* = 12.7881 (18) Å
*c* = 19.479 (3) Å
β = 126.735 (2)°
*V* = 4824.8 (12) Å^3^
*Z* = 8
Mo *K*α radiation
μ = 0.06 mm^−1^
*T* = 291 (2) K
0.45 × 0.34 × 0.30 mm

#### Data collection

Bruker SMART 1K CCD diffractometer
Absorption correction: none
12048 measured reflections
4242 independent reflections
2237 reflections with *I* > 2σ(*I*)
*R*_int_ = 0.036

#### Refinement

*R*[*F* ^2^ > 2σ(*F* ^2^)] = 0.051
*wR*(*F* ^2^) = 0.161
*S* = 1.02
4242 reflections
252 parameters
H-atom parameters constrained
Δρ_max_ = 0.18 e Å^−3^
Δρ_min_ = −0.17 e Å^−3^

### 

Data collection: *SMART* (Bruker, 2003 ▶); cell refinement: *SAINT* (Bruker, 2003 ▶); data reduction: *SAINT*; program(s) used to solve structure: *SHELXS97* (Sheldrick, 2008 ▶); program(s) used to refine structure: *SHELXL97* (Sheldrick, 2008 ▶); molecular graphics: *ORTEP-3 for Windows* (Farrugia, 1997 ▶); software used to prepare material for publication: *publCIF* (Westrip, 2008 ▶).

## Supplementary Material

Crystal structure: contains datablocks I, global. DOI: 10.1107/S160053680802076X/bi2287sup1.cif

Structure factors: contains datablocks I. DOI: 10.1107/S160053680802076X/bi2287Isup2.hkl

Additional supplementary materials:  crystallographic information; 3D view; checkCIF report

## Figures and Tables

**Table 1:** Hydrogen-bond geometry (Å, °)

*D*—H⋯*A*	*D*—H	H⋯*A*	*D*⋯*A*	*D*—H⋯*A*
N1—H1*A*⋯N1^i^	0.86	2.03	2.882 (4)	171
N2—H2*A*⋯N2^i^	0.86	2.05	2.910 (3)	175

Symmetry code: (i) 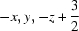.

**Fig. 1.**
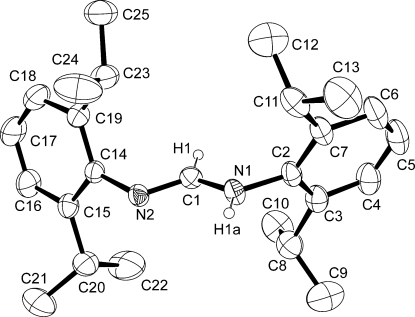
Molecular structure showing displacement ellipsoids at the 30% probability level for non-H atoms. H atoms bound to C (except for H1) are omitted, and only one of the disordered H atoms (H1A & H1B) is shown.

**Fig. 2.**
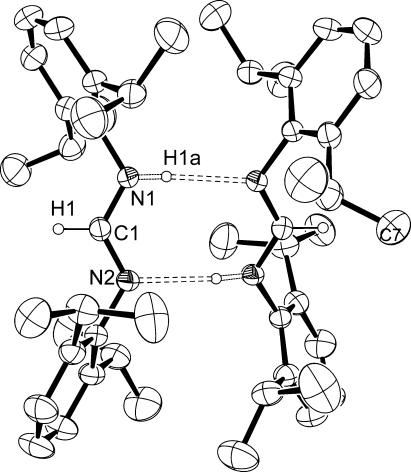
A plot of the hydrogen-bonded dimer in the title compound, showing displacement ellipsoids at the 30% probability level for non-H atoms. H atoms bound to C (except for H1) are omitted, and only one of the disordered H atoms (H1A & H1B) is shown.

## supplementary crystallographic information

### Comment

Crystals of the title compound were grown from toluene solution and were found to crystallize in the monoclinic space group *C*2/*c*, different from the previously published polymorph which crystallizes in the orthorhombic space group *C*222_1_ (Stibrany & Potenza, 2006). The molecule crystallizes in the *E*-*anti* configuration (Perrin, 1991), with tautomeric disorder of the N-bonded H atoms. The molecules form hydrogen-bonded dimers with N···N distances of 2.882 (4) and 2.910 (3) Å (Table 1). These distances are slightly shorter than that seen in the orthorhombic polymorph (2.947 Å). The two core amidine (NCNH) fragments are non-coplanar as a result of interaction between the sterically bulky 2,6-diisopropylphenyl fragments. The N1—C(1) (1.313 (3) Å) and N2—C1 (1.311 (3) Å) distances are similar in length, whereas in the orthorhombic polymorph there are distinct imine (1.288 Å) and amine (1.325 Å) functionalities.

### Experimental

The title compound was prepared according to the literature procedure (Krahulic *et al.*, 2005). Crystals were grown by evaporation of a toluene solution at room temperature.

### Refinement

H atoms bonded to C and N atoms were refined in geometrically idealized positions with the riding-model approximation. The difference map showed equivalent electron density for the H atoms bonded to the formamidine N atoms. Thus, the H atom was refined as disordered over two positions, each with site occupancy factor 0.5.

### Crystal data

**Table:**

C_25_H_36_N_2_	*F*_000_ = 1600
*M**_r_* = 364.56	*D*_x_ = 1.004 Mg m^−^^3^
Monoclinic, *C*2/*c*	Mo *K*α radiation λ = 0.71073 Å
Hall symbol: -C 2yc	Cell parameters from 2205 reflections
*a* = 24.169 (4) Å	θ = 2.6–21.8º
*b* = 12.7881 (18) Å	µ = 0.06 mm^−^^1^
*c* = 19.479 (3) Å	*T* = 291 (2) K
β = 126.735 (2)º	Block, colourless
*V* = 4824.8 (12) Å^3^	0.45 × 0.34 × 0.30 mm
*Z* = 8	

### Data collection

**Table:**

Bruker SMART 1K CCD diffractometer	2237 reflections with *I* > 2σ(*I*)
Radiation source: fine-focus sealed tube	*R*_int_ = 0.036
Monochromator: graphite	θ_max_ = 25.0º
*T* = 291(2) K	θ_min_ = 2.1º
φ and ω scans	*h* = −28→28
Absorption correction: none	*k* = −7→15
12048 measured reflections	*l* = −23→23
4242 independent reflections	

### Refinement

**Table:**

Refinement on *F*^2^	Secondary atom site location: difference Fourier map
Least-squares matrix: full	Hydrogen site location: inferred from neighbouring sites
*R*[*F*^2^ > 2σ(*F*^2^)] = 0.051	H-atom parameters constrained
*wR*(*F*^2^) = 0.161	*w* = 1/[σ^2^(*F*_o_^2^) + (0.065*P*)^2^ + 2.1873*P*] where *P* = (*F*_o_^2^ + 2*F*_c_^2^)/3
*S* = 1.02	(Δ/σ)_max_ < 0.001
4242 reflections	Δρ_max_ = 0.18 e Å^−^^3^
252 parameters	Δρ_min_ = −0.17 e Å^−^^3^
Primary atom site location: structure-invariant direct methods	Extinction correction: none

### Special details

**Table:**

Geometry. All e.s.d.'s (except the e.s.d. in the dihedral angle between two l.s. planes) are estimated using the full covariance matrix. The cell e.s.d.'s are taken into account individually in the estimation of e.s.d.'s in distances, angles and torsion angles; correlations between e.s.d.'s in cell parameters are only used when they are defined by crystal symmetry. An approximate (isotropic) treatment of cell e.s.d.'s is used for estimating e.s.d.'s involving l.s. planes.
Refinement. Refinement of *F*^2^ against ALL reflections. The weighted *R*-factor *wR* and goodness of fit *S* are based on *F*^2^, conventional *R*-factors *R* are based on *F*, with *F* set to zero for negative *F*^2^. The threshold expression of *F*^2^ > σ(*F*^2^) is used only for calculating *R*-factors(gt) *etc*. and is not relevant to the choice of reflections for refinement. *R*-factors based on *F*^2^ are statistically about twice as large as those based on *F*, and *R*- factors based on ALL data will be even larger.

### Fractional atomic coordinates and isotropic or equivalent isotropic displacement parameters (Å^2^)

**Table:**

	*x*	*y*	*z*	*U*_iso_*/*U*_eq_	Occ. (<1)
N2	0.02590 (9)	0.18752 (13)	0.69910 (11)	0.0465 (5)	
H2A	0.0113	0.1919	0.7299	0.056*	0.50
N1	0.00186 (10)	0.01071 (13)	0.67743 (12)	0.0502 (5)	
H1A	−0.0042	0.0087	0.7167	0.060*	0.50
C2	−0.00791 (13)	−0.08218 (17)	0.63024 (16)	0.0509 (6)	
C1	0.01996 (11)	0.09893 (17)	0.66139 (14)	0.0468 (6)	
H1	0.0290	0.0985	0.6212	0.056*	
C14	0.05632 (11)	0.27673 (17)	0.68974 (14)	0.0463 (6)	
C15	0.01510 (13)	0.36571 (18)	0.64789 (15)	0.0554 (6)	
C19	0.12706 (12)	0.27631 (19)	0.72640 (16)	0.0569 (6)	
C7	0.03995 (14)	−0.16434 (18)	0.67252 (17)	0.0607 (7)	
C3	−0.06573 (14)	−0.0910 (2)	0.54441 (16)	0.0612 (7)	
C11	0.10007 (15)	−0.1590 (2)	0.76716 (17)	0.0705 (8)	
H11A	0.0904	−0.1015	0.7917	0.085*	
C8	−0.12183 (15)	−0.0076 (2)	0.50015 (18)	0.0746 (8)	
H8A	−0.1093	0.0474	0.5422	0.090*	
C6	0.03099 (18)	−0.2520 (2)	0.6239 (2)	0.0797 (9)	
H6A	0.0627	−0.3064	0.6499	0.096*	
C4	−0.07140 (17)	−0.1813 (2)	0.50041 (19)	0.0781 (9)	
H4A	−0.1090	−0.1884	0.4432	0.094*	
C20	−0.06095 (13)	0.3655 (2)	0.60888 (18)	0.0692 (8)	
H20A	−0.0673	0.3122	0.6397	0.083*	
C16	0.04601 (16)	0.4528 (2)	0.64050 (19)	0.0776 (8)	
H16A	0.0196	0.5120	0.6120	0.093*	
C17	0.11450 (18)	0.4525 (2)	0.6744 (2)	0.0902 (10)	
H17A	0.1341	0.5113	0.6688	0.108*	
C23	0.17226 (13)	0.1832 (2)	0.77905 (18)	0.0719 (8)	
H23A	0.1449	0.1200	0.7502	0.086*	
C18	0.15455 (15)	0.3660 (2)	0.7168 (2)	0.0803 (9)	
H18A	0.2011	0.3673	0.7396	0.096*	
C5	−0.0236 (2)	−0.2596 (2)	0.5387 (2)	0.0874 (10)	
H5A	−0.0279	−0.3179	0.5072	0.105*	
C25	0.23851 (14)	0.1727 (3)	0.7863 (2)	0.0935 (10)	
H25A	0.2276	0.1749	0.7302	0.140*	
H25B	0.2605	0.1074	0.8133	0.140*	
H25C	0.2691	0.2293	0.8201	0.140*	
C13	0.10778 (19)	−0.2581 (3)	0.8163 (2)	0.1084 (12)	
H13A	0.1433	−0.2478	0.8764	0.163*	
H13B	0.1200	−0.3158	0.7963	0.163*	
H13C	0.0649	−0.2730	0.8071	0.163*	
C12	0.16764 (18)	−0.1332 (3)	0.7815 (2)	0.1097 (12)	
H12A	0.1630	−0.0682	0.7539	0.165*	
H12B	0.1788	−0.1879	0.7579	0.165*	
H12C	0.2038	−0.1273	0.8418	0.165*	
C10	−0.12660 (19)	0.0421 (3)	0.4259 (2)	0.1045 (11)	
H10A	−0.0823	0.0697	0.4461	0.157*	
H10B	−0.1599	0.0976	0.4021	0.157*	
H10C	−0.1406	−0.0097	0.3827	0.157*	
C24	0.18908 (18)	0.1848 (3)	0.8679 (2)	0.1184 (14)	
H24A	0.1470	0.1816	0.8627	0.178*	
H24B	0.2133	0.2482	0.8968	0.178*	
H24C	0.2175	0.1258	0.9002	0.178*	
C21	−0.08718 (19)	0.4678 (3)	0.6196 (3)	0.1195 (13)	
H21A	−0.0574	0.4892	0.6785	0.179*	
H21B	−0.1332	0.4581	0.6026	0.179*	
H21C	−0.0875	0.5207	0.5844	0.179*	
C9	−0.19216 (18)	−0.0515 (3)	0.4695 (3)	0.1260 (14)	
H9A	−0.1900	−0.0740	0.5181	0.189*	
H9B	−0.2038	−0.1099	0.4321	0.189*	
H9C	−0.2267	0.0018	0.4392	0.189*	
C22	−0.10306 (17)	0.3317 (4)	0.5178 (2)	0.1415 (17)	
H22A	−0.0855	0.2666	0.5135	0.212*	
H22B	−0.1007	0.3839	0.4842	0.212*	
H22C	−0.1502	0.3226	0.4969	0.212*	

### Atomic displacement parameters (Å^2^)

**Table:**

	*U*^11^	*U*^22^	*U*^33^	*U*^12^	*U*^13^	*U*^23^
N2	0.0542 (12)	0.0376 (11)	0.0561 (12)	−0.0011 (9)	0.0376 (10)	−0.0006 (9)
N1	0.0740 (14)	0.0368 (11)	0.0566 (12)	0.0004 (10)	0.0481 (11)	−0.0005 (9)
C2	0.0741 (17)	0.0376 (13)	0.0615 (16)	−0.0029 (12)	0.0516 (15)	−0.0020 (12)
C1	0.0513 (14)	0.0483 (14)	0.0475 (13)	0.0015 (11)	0.0330 (12)	0.0025 (12)
C14	0.0511 (14)	0.0420 (13)	0.0495 (13)	−0.0059 (11)	0.0322 (12)	−0.0040 (11)
C15	0.0612 (16)	0.0429 (14)	0.0589 (16)	−0.0023 (12)	0.0342 (14)	0.0009 (12)
C19	0.0550 (16)	0.0551 (15)	0.0616 (16)	−0.0068 (13)	0.0355 (13)	−0.0058 (13)
C7	0.089 (2)	0.0415 (14)	0.0725 (18)	0.0033 (14)	0.0599 (17)	0.0001 (13)
C3	0.0817 (19)	0.0557 (16)	0.0582 (17)	−0.0070 (14)	0.0483 (16)	−0.0065 (14)
C11	0.090 (2)	0.0576 (17)	0.074 (2)	0.0200 (15)	0.0539 (18)	0.0119 (15)
C8	0.078 (2)	0.080 (2)	0.0591 (17)	−0.0008 (17)	0.0373 (16)	−0.0067 (15)
C6	0.120 (3)	0.0427 (16)	0.100 (3)	0.0088 (16)	0.079 (2)	−0.0006 (16)
C4	0.106 (2)	0.069 (2)	0.0666 (19)	−0.0134 (18)	0.0555 (18)	−0.0150 (17)
C20	0.0616 (17)	0.0590 (17)	0.079 (2)	0.0106 (14)	0.0379 (16)	0.0132 (15)
C16	0.086 (2)	0.0506 (16)	0.095 (2)	−0.0034 (15)	0.0534 (19)	0.0093 (16)
C17	0.093 (2)	0.0605 (19)	0.125 (3)	−0.0231 (18)	0.069 (2)	0.003 (2)
C23	0.0534 (16)	0.0719 (18)	0.083 (2)	0.0030 (14)	0.0369 (15)	0.0051 (16)
C18	0.0636 (18)	0.076 (2)	0.101 (2)	−0.0171 (16)	0.0493 (18)	−0.0044 (18)
C5	0.139 (3)	0.0538 (19)	0.096 (3)	−0.009 (2)	0.084 (2)	−0.0201 (18)
C25	0.0622 (18)	0.117 (3)	0.094 (2)	0.0095 (18)	0.0426 (17)	−0.009 (2)
C13	0.146 (3)	0.089 (2)	0.107 (3)	0.024 (2)	0.085 (3)	0.034 (2)
C12	0.099 (3)	0.127 (3)	0.105 (3)	0.010 (2)	0.062 (2)	0.019 (2)
C10	0.127 (3)	0.099 (3)	0.088 (2)	0.021 (2)	0.064 (2)	0.022 (2)
C24	0.097 (2)	0.168 (4)	0.099 (3)	0.056 (3)	0.063 (2)	0.052 (3)
C21	0.109 (3)	0.097 (3)	0.158 (4)	0.034 (2)	0.084 (3)	0.006 (3)
C9	0.096 (3)	0.149 (4)	0.137 (3)	−0.001 (3)	0.071 (3)	0.009 (3)
C22	0.067 (2)	0.214 (5)	0.096 (3)	0.005 (3)	0.023 (2)	−0.040 (3)

### Geometric parameters (Å, °)

**Table:**

N2—C1	1.310 (3)	C16—H16A	0.930
N2—C14	1.427 (3)	C17—C18	1.373 (4)
N2—H2A	0.860	C17—H17A	0.930
N1—C1	1.313 (3)	C23—C24	1.523 (4)
N1—C2	1.432 (3)	C23—C25	1.527 (4)
N1—H1A	0.860	C23—H23A	0.980
C2—C3	1.402 (3)	C18—H18A	0.930
C2—C7	1.407 (3)	C5—H5A	0.930
C1—H1	0.930	C25—H25A	0.960
C14—C19	1.404 (3)	C25—H25B	0.960
C14—C15	1.407 (3)	C25—H25C	0.960
C15—C16	1.395 (3)	C13—H13A	0.960
C15—C20	1.512 (3)	C13—H13B	0.960
C19—C18	1.392 (3)	C13—H13C	0.960
C19—C23	1.524 (3)	C12—H12A	0.960
C7—C6	1.398 (3)	C12—H12B	0.960
C7—C11	1.520 (4)	C12—H12C	0.960
C3—C4	1.395 (4)	C10—H10A	0.960
C3—C8	1.523 (4)	C10—H10B	0.960
C11—C12	1.519 (4)	C10—H10C	0.960
C11—C13	1.531 (4)	C24—H24A	0.960
C11—H11A	0.980	C24—H24B	0.960
C8—C10	1.520 (4)	C24—H24C	0.960
C8—C9	1.532 (4)	C21—H21A	0.960
C8—H8A	0.980	C21—H21B	0.960
C6—C5	1.373 (4)	C21—H21C	0.960
C6—H6A	0.930	C9—H9A	0.960
C4—C5	1.365 (4)	C9—H9B	0.960
C4—H4A	0.930	C9—H9C	0.960
C20—C22	1.487 (4)	C22—H22A	0.960
C20—C21	1.522 (4)	C22—H22B	0.960
C20—H20A	0.980	C22—H22C	0.960
C16—C17	1.367 (4)		
			
C1—N2—C14	120.78 (18)	C19—C23—C25	114.9 (2)
C1—N2—H2A	119.6	C24—C23—H23A	107.0
C14—N2—H2A	119.6	C19—C23—H23A	107.0
C1—N1—C2	120.68 (18)	C25—C23—H23A	107.0
C1—N1—H1A	119.7	C17—C18—C19	121.5 (3)
C2—N1—H1A	119.7	C17—C18—H18A	119.3
C3—C2—C7	121.5 (2)	C19—C18—H18A	119.3
C3—C2—N1	119.7 (2)	C4—C5—C6	119.7 (3)
C7—C2—N1	118.8 (2)	C4—C5—H5A	120.2
N2—C1—N1	123.3 (2)	C6—C5—H5A	120.2
N2—C1—H1	118.3	C23—C25—H25A	109.5
N1—C1—H1	118.3	C23—C25—H25B	109.5
C19—C14—C15	121.5 (2)	H25A—C25—H25B	109.5
C19—C14—N2	119.7 (2)	C23—C25—H25C	109.5
C15—C14—N2	118.7 (2)	H25A—C25—H25C	109.5
C16—C15—C14	117.9 (2)	H25B—C25—H25C	109.5
C16—C15—C20	121.0 (2)	C11—C13—H13A	109.5
C14—C15—C20	121.1 (2)	C11—C13—H13B	109.5
C18—C19—C14	117.5 (2)	H13A—C13—H13B	109.5
C18—C19—C23	121.8 (2)	C11—C13—H13C	109.5
C14—C19—C23	120.6 (2)	H13A—C13—H13C	109.5
C6—C7—C2	117.5 (3)	H13B—C13—H13C	109.5
C6—C7—C11	120.4 (3)	C11—C12—H12A	109.5
C2—C7—C11	122.1 (2)	C11—C12—H12B	109.5
C4—C3—C2	117.4 (3)	H12A—C12—H12B	109.5
C4—C3—C8	120.3 (3)	C11—C12—H12C	109.5
C2—C3—C8	122.2 (2)	H12A—C12—H12C	109.5
C12—C11—C7	112.1 (2)	H12B—C12—H12C	109.5
C12—C11—C13	110.4 (3)	C8—C10—H10A	109.5
C7—C11—C13	113.1 (3)	C8—C10—H10B	109.5
C12—C11—H11A	107.0	H10A—C10—H10B	109.5
C7—C11—H11A	107.0	C8—C10—H10C	109.5
C13—C11—H11A	107.0	H10A—C10—H10C	109.5
C10—C8—C3	111.5 (3)	H10B—C10—H10C	109.5
C10—C8—C9	110.4 (3)	C23—C24—H24A	109.5
C3—C8—C9	111.6 (3)	C23—C24—H24B	109.5
C10—C8—H8A	107.7	H24A—C24—H24B	109.5
C3—C8—H8A	107.7	C23—C24—H24C	109.5
C9—C8—H8A	107.7	H24A—C24—H24C	109.5
C5—C6—C7	121.6 (3)	H24B—C24—H24C	109.5
C5—C6—H6A	119.2	C20—C21—H21A	109.5
C7—C6—H6A	119.2	C20—C21—H21B	109.5
C5—C4—C3	122.1 (3)	H21A—C21—H21B	109.5
C5—C4—H4A	118.9	C20—C21—H21C	109.5
C3—C4—H4A	118.9	H21A—C21—H21C	109.5
C22—C20—C15	111.0 (2)	H21B—C21—H21C	109.5
C22—C20—C21	111.7 (3)	C8—C9—H9A	109.5
C15—C20—C21	114.2 (2)	C8—C9—H9B	109.5
C22—C20—H20A	106.4	H9A—C9—H9B	109.5
C15—C20—H20A	106.4	C8—C9—H9C	109.5
C21—C20—H20A	106.4	H9A—C9—H9C	109.5
C17—C16—C15	121.1 (3)	H9B—C9—H9C	109.5
C17—C16—H16A	119.5	C20—C22—H22A	109.5
C15—C16—H16A	119.5	C20—C22—H22B	109.5
C16—C17—C18	120.5 (3)	H22A—C22—H22B	109.5
C16—C17—H17A	119.7	C20—C22—H22C	109.5
C18—C17—H17A	119.7	H22A—C22—H22C	109.5
C24—C23—C19	110.5 (2)	H22B—C22—H22C	109.5
C24—C23—C25	110.1 (2)		

### Hydrogen-bond geometry (Å, °)

**Table:**

*D*—H···*A*	*D*—H	H···*A*	*D*···*A*	*D*—H···*A*
N1—H1A···N1^i^	0.86	2.03	2.882 (4)	171
N2—H2A···N2^i^	0.86	2.05	2.910 (3)	175

Symmetry codes: (i) −*x*, *y*, −*z*+3/2.
